# Individualised, flexible postnatal care: a feasibility study for a randomised controlled trial

**DOI:** 10.1186/s12913-014-0569-2

**Published:** 2014-11-25

**Authors:** Della A Forster, Tracey L Savage, Helen L McLachlan, Lisa Gold, Tanya Farrell, Jo Rayner, Jane Yelland, Bree Rankin, Belinda Lovell

**Affiliations:** Judith Lumely Centre, La Trobe University, 215 Franklin St, Melbourne, 3000 Australia; The Royal Women’s Hospital, Locked Bag 300, Cnr Grattan St and Flemington Rd, Parkville, 3052 Australia; School of Nursing and Midwifery, La Trobe University, Melbourne Campus, Kingsbury Drive, Bundoora, Vic 3086 Australia; Deakin Health Economics, Deakin University, 221 Burwood Highway, Burwood, 3125 Australia; Healthy Mothers Healthy Families Research Group, Murdoch Childrens Research Institute, 50 Flemington Road, Parkville, Victoria 3052 Australia; Drug Strategy Analysis Unit, Population 846 Health Division, Department of Health and Ageing, Canberra, Australia; Yale New Haven Hospital, 20 York Street, New Haven, CT 06510 USA

**Keywords:** Postnatal care, Domiciliary care, Individualised care, Early discharge, Postnatal preparation

## Abstract

**Background:**

Postnatal care in hospital is often provided using defined care pathways, with limited opportunity for more refined and individualised care. We explored whether a tertiary maternity service could provide flexible, individualised early postnatal care for women in a dynamic and timely manner, and if this approach was acceptable to women.

**Methods:**

A feasibility study was designed to inform a future randomised controlled trial to evaluate an alternative approach to postnatal care. English-speaking women at low risk of medical complications were recruited around 26 weeks gestation to explore their willingness to participate in a study of a new, flexible model of care that involved antenatal planning for early postpartum discharge with additional home-based postnatal care. The earlier women were discharged from hospital, the more home-based visits they were eligible to receive. Program uptake was measured, women’s views obtained by a postal survey sent at eight weeks postpartum and clinical data collected from medical records.

**Results:**

Study uptake was 39% (109/277 approached). Most women (n=103) completed a postnatal care plan during pregnancy; 17% planned to leave hospital within 12 hours of giving birth and 36% planned to stay 48 hours. At eight weeks postpartum most women (90%) were positive about the concept and 88% would opt for the same program again. Of the 28% who stayed in hospital for the length they had planned, less than half (43%) received the appropriate number of home visits, and only 41% were given an option for the timing of the visit. Most (62%) stayed in hospital longer than planned (probably due to clinical complications); 11% stayed shorter than planned.

**Conclusions:**

Women were very positive about individualised postnatal care planning that commenced during pregnancy. Given the hospital stay may be impacted by clinical factors, individualised care planning needs to continue into the postnatal period to take into account circumstances which cannot be planned for during pregnancy. However, individualised care planning during the postnatal period which incorporates a high level of flexibility may be challenging for organisations to manage and implement, and a randomised controlled trial of such an approach may not be feasible.

## Background

*Whether postnatal care is provided in hospital or in the woman’s home, it is imperative that the care provided is of the highest standard and meets the needs of the individual* [1] *p21.*

In Australia, increasing birth numbers, decreasing length of hospital postnatal stay and uncertainty regarding the optimal content and location of care provision are challenging issues in postnatal care. There is limited evidence about the impact of how care is provided in the early postnatal period - for example, impact(s) on the physical and/or emotional health of the mother or the baby.

In Victoria, Australia, there has been increasing numbers of women giving birth [2] and a subsequent lack of physical space to provide hospital postnatal care for women. In response many hospitals have needed to discharge women much earlier than previously (with little evaluation of the effect of these changes), and the length of postnatal hospital stay has declined dramatically since the 1980’s. In 2009, over one-third (39%) of all women stayed in hospital two days or less, compared with 4% in 1985 [2]. In 2009–10, the average length of stay for a public hospital birth episode was two days for an uncomplicated vaginal birth and four days for a caesarean section without major complications [3]. Following discharge from the public hospital system in Victoria, (where two thirds of all women have maternity care [3]) most new mothers receive the offer of at least one domiciliary midwife visit. The recently released *Postnatal Care Program Guidelines for Victorian Health Services* state that “as a minimum requirement, following discharge, public health services should offer women at least one postnatal visit in her home”, and that “additional home visits [be] provided on the basis of individual clinical and psychosocial needs” [1] p21. A statewide review of domiciliary care in Victoria found that overall, the median number of home visits for primiparous women was two (range one to four) and for multiparous women, one (range one to three) [4].

The Cochrane review of early postnatal discharge from hospital for healthy mothers and term infants concluded that early discharge does not appear to have adverse effects on breastfeeding or maternal depression when accompanied by a policy of offering women at least one nurse-midwife home visit post discharge [5]. However, the authors concluded that large well-designed trials of early discharge programs are needed, incorporating process evaluation to assess the uptake of co-interventions, and using standardised approaches to outcome assessment.

In terms of women’s views of care, the component of maternity care which women consistently rate less favourably is postnatal care. This has been reported in Australia [6,7] and elsewhere [8-10]. Only half of the women participating in a state-wide survey of new mothers in Victoria in 2004 rated their postnatal care as ‘very good’, compared to 67% and 72% who rated their antenatal and intrapartum care respectively as ‘very good’ [7]. Various factors were associated with satisfaction with care, and length of the hospital stay following birth was one of these; staying in hospital for one to two days was associated with less positive ratings of care compared with staying five days or more [7].

In 2006 we conducted focus groups in rural and metropolitan Victoria to gain more in-depth information on women’s views of postnatal care and in particular to gain an understanding about their views on earlier postpartum discharge home. Fifty-two people participated in eight focus groups and four interviews [11]. This included eight pregnant women, of whom seven were pregnant with their first baby; 42 women who were in the postpartum period (some up to 12 months after the birth of their baby); and two partners. Women were generally concerned about the safety of their new baby, and lacked confidence in themselves as new mothers regarding their ability to care for their baby. There was a prevailing view that the physical presence and availability of professional support helped alleviate these concerns, and this was especially the case for women having a first baby. Women had anxieties and fears around early parenting and their changing role. Consistent with these views, many women were concerned about any moves to make the postnatal hospital stay shorter, especially for first time mothers. We concluded that any changes to care provision should be evaluated, that women’s views should be taken into account, and that where possible providers should ensure that care is individualised to address each woman’s/family’s particular concerns [11].

Providers also have concerns about postnatal care provision. A Victorian state-wide review of hospital postnatal care based on the views of care providers found that there were a number of barriers to postnatal care provision including the busyness of postnatal wards, inadequate staffing numbers, and priority being given to other episodes of care [12,13]. The review highlighted a great diversity in the provision of postnatal care across the State in relation to models of care, staffing arrangements, and routine practices [13]. There was a strong sense among care providers that the provision of hospital based postnatal care is considered a lower priority than the other episodes of maternity care.

Given the context of maternity care in Victoria and elsewhere, it is important to consider how best to identify which women could receive more of their postnatal care at home, and what this care should involve. The provision of postnatal care at home following hospital discharge in the Australian context has had limited evaluation, although there are indications that women rate the care that they receive at home more highly than they do the care they receive in hospital [14-16]. A Victorian statewide review of home-based (domiciliary) care explored the structure and organisation of this care [4], but there is little data on the outcomes of home-based care, nor the views and experiences of women and care providers.

Although both the Victorian guidelines on postnatal care and the NICE guidelines on the routine care of postnatal women and their babies [17] suggest all care should be individualised, we identified no evidence regarding whether or not a more individualised approach to postnatal care is feasible or practicable from an organisational perspective, and whether the balance of care provided in hospital and at home can be optimised for each woman. In light of this, and considering that a number of Victorian maternity services were already moving towards very early postnatal discharge with little or no evaluation, we undertook a study to systematically pilot a new approach to postnatal care to provide a framework for further evaluation. The Postnatal individualised Care (PinC) Program was a feasibility study designed to inform a future randomised controlled trial which would evaluate an alternative approach to postnatal care. The aim was to explore the workability, costs and acceptability (to women and to care providers) of a new approach to early postnatal care, in particular the ability of a tertiary maternity service to provide flexible, individualised early postnatal care for women in a dynamic and timely manner.

Two aspects of this pilot ran simultaneously, a pilot of the intervention with women (the focus of this paper), along with focus groups and interviews with staff. This paper describes the alternative approach to early postnatal care we developed and presents data on whether women received the care we planned to be provided and what women’s experiences were, as well as discussing the organisational factors that may have impacted on the implementation of the intervention. Staff views and costing data will be reported elsewhere.

## Methods

### Study design

A model of individualised postnatal care was developed and piloted. Eligible women were recruited to explore whether an individualised plan for postnatal care could be established in consultation with women at around 26 weeks gestation, and that it would be able to evolve to meet women’s changing needs. This gestation was chosen as one of only two time points in pregnancy that all women attend the hospital for care (the other being the initial booking appointment), therefore considered the best option. No control group was included, as the aim was to determine the feasibility of the intervention within a metropolitan tertiary maternity hospital.

### Participants

Women attending the Royal Women’s Hospital in Melbourne (a large tertiary referral hospital with over 7,000 births per year) for pregnancy care as public patients were eligible. Women were excluded if they: were receiving birth centre care (associated with a short length of stay); had medical or obstetric risk factors that may have made them ineligible for short hospital stays; were less than 24 weeks gestation (although these women could be eligible later); were more than 30 weeks gestation (the intervention involved antenatal preparation, therefore women >30 weeks had inadequate time for antenatal preparation); were non-English speaking (NES); lived outside the hospital domiciliary area; were less than 16 years of age; or had significant social and psychological issues or risks. Women with planned or unplanned caesarean births were *not* excluded.

### Recruitment

Brochures describing the program were available in all antenatal clinic waiting areas. Recruitment was conducted by a research midwife (TS). We aimed to approach all eligible women attending a selection of clinics that were chosen to be representative of all clinics held during the recruitment period, to ensure diversity in the sample. Women who agreed to participate provided written informed consent.

### Sample size

We aimed to recruit up to 200 women, with no less than 100. These numbers were chosen not to show statistically significant differences in any outcomes, but to establish: the feasibility of undertaking this type of individualised, flexible care in a tertiary facility; the proportion of women willing to agree to participate in such a study; baseline data about likely cross-over in a model such as this; to explore issues in costing this type of care; and to identify the processes and education that would need to be put in place to implement a much larger version of this model.

### The intervention

**Figure 1:**
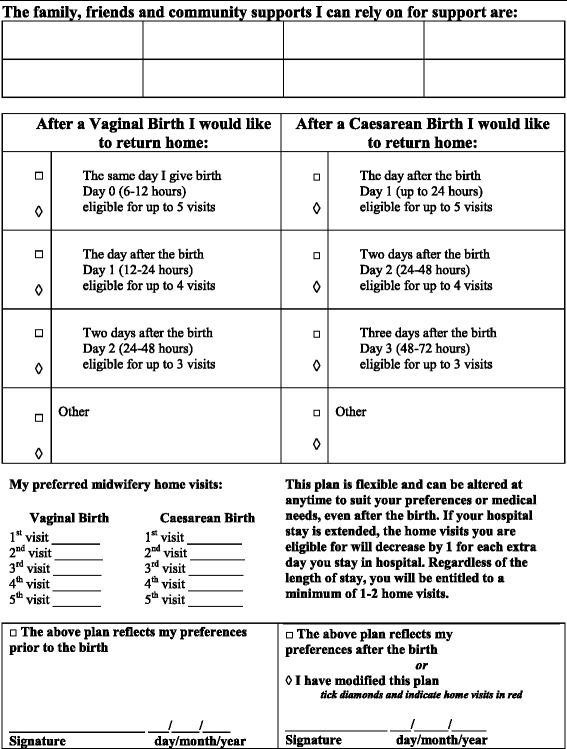
**PinC program individualised plan of postnatal care.**

The underlying idea being tested was that women could ‘trade’ time in hospital in the postnatal period for extra home visits following discharge, at a time and schedule that suited them. For example, if a woman went home within 12 hours of a normal birth she would be eligible for up to five home visits (as opposed to the more usual single visit) (Figure 1). In theory, the woman could choose to have a visit on the day of discharge, and even to have two visits on one day. That is, we aimed to test the ability of the organisation to provide postnatal care that was individualised and that was responsive to women’s needs, provided in a timely manner, and in the context of the unpredictability of births. In addition, we wanted this to be a result of careful planning on the part of the woman and her family, not an ad hoc decision after the birth.

Women had a preliminary discussion on preparation for early discharge at the time of study recruitment (Table 1), to explore the concept of a flexible approach to early discharge and postnatal care at home, and to begin to consider what options would be most appropriate to their situation. They then had an additional appointment with the study midwife when attending for their 36 weeks gestation antenatal visit, to create their individualised plan of postnatal care. This antenatal planning for discharge was the key intervention, allowing the opportunity to ensure that the supports individual women might need at home were in place prior to labour and birth, well ahead of postnatal discharge wherever possible. Labour, birth and postnatal hospital care were provided as usual.

**Table 1:** **PinC individualised plan of postnatal care**

**PinC Program pilot 36 week dialogue: a guide**	
**Introduction:**	This meeting is to discuss your preferred plan of care. You have had a couple of months to think about what you would like and I am interested to hear what you like to happen after you have your baby.
**Assess personal support:**	Have you had a chance to think about the supports you have at home? What family and friends do you intend to rely on? Who can help you with cooking, cleaning and shopping? *Fill in details on plan*
**Community Support:**	We will provide you with a direct phone number to speak with a midwife from your team once you go home. This number will be 24 hours a day for any question or concerns while you are at home. Your midwife may have already mentioned other community supports at your antenatal visits. Can you tell what community supports you are aware of? *Fill in details on plan, prompt with relevant brochures if necessary*
**Length of Stay:**	Provided you and your baby are both healthy and medically cleared for discharge, how long do you think you would like to stay in hospital after the birth? *Fill in details on plan*
**Home visits:**	What is your preference for timing of home visits by midwives? *Fill in details on plan ie: day 0, day 1, day 2 for home visits*
**Conclude visit:**	Do you have any questions? *Re-present program brochure given at time of recruitment. Participant to sign plan.* For further questions or concerns, use the contact details on the brochure.

The postnatal plan, which detailed women’s intended length of hospital stay and preference for scheduling of home visits, was documented on the ‘PinC Program Individualised plan of postnatal care’ form (Figure 1), which was printed on bright pink paper and filed at the front of the woman’s medical record. The plan could be modified at any time, including after the birth, allowing for a flexible length of stay for women. The aim was that at the time of discharge (as specified by the woman), care providers would arrange for the appropriate home visits to be provided at mutually agreeable times as per the agreed plan.

### Data collection and analysis

Demographic data were collected by questionnaire, completed by women at recruitment. Additional data were collected from the postnatal plans completed at 36 weeks and by a postal questionnaire sent to women eight weeks postpartum. The questionnaire explored women’s views and experiences of the new model, and included mainly structured questions, with opportunities for women to comment further. The questionnaire was based on survey instruments used previously [18,19] and included questions specific to the project. A reminder letter was sent to all participants two weeks after the original mail-out and a phone call reminder after four weeks. Medical data pertaining to relevant readmissions were abstracted from the medical record a month after the birth. The number of women who were excluded and the proportion who consented to participate was documented to determine study uptake.

Data from questionnaires, medical records and postnatal individual plans were entered into a Microsoft Access database [20] and analysed using STATA version 8 [21]. Analysis for pre-coded responses was undertaken using descriptive statistics.

Women’s responses (comments) to open-ended questions were analysed inductively and grouped into analytical descriptive categories [22]. Insights into women’s experiences of the new model of care emerged from the comments they provided and direct quotes are used to illustrate the findings. As stated in the consent forms that were used, no information provided enables identification of individual women – the three identifiers used for direct quotes are age, type of birth and whether it is a first baby. Given there are over 7,000 births per year at the study hospital this was considered sufficiently anonymising [23]. This aspect of the data analysis was done in accordance with the RATS guidelines [24]; that is, ensuring the Relevance of the research question, using an Appropriate method to collect the data, maintaining Transparent procedures (such as sampling, subject recruitment, ethics), and a Sound interpretive approach.

Ethical approval was provided by the Human Research Ethics Committee of La Trobe University (HEC 07–78) and the Royal Women’s Hospital Research and Ethics Committees (Project 07–20).

## Results

### Participants

**Figure 2:**
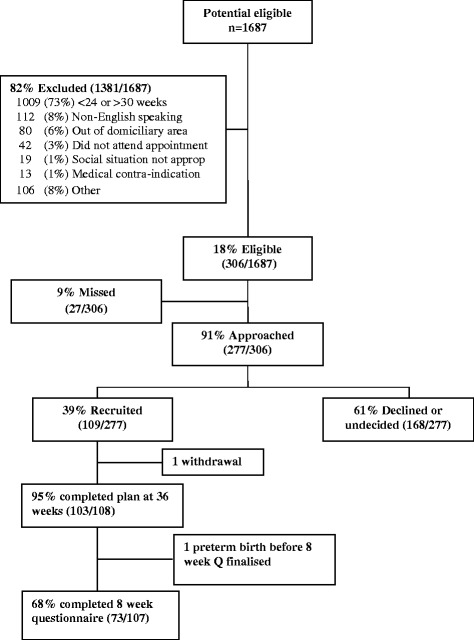
**Participant recruitment.**

Women were recruited between September 2007 and January 2008. Of the 1,687 potentially eligible women attending the 69 antenatal clinics attended by the research midwife, 82% were excluded after a brief review of the medical record, mainly due to gestation outside the eligible range (Figure 2). Of the 306 eligible women, 91% (277) were approached and of those, 109 agreed to participate, representing 39% of the eligible women approached. One participant withdrew later, decreasing the sample size to 108, and another did not consent to medical record access, so data on birth outcomes and hospital care are presented for 107 women.

The majority of participants were married (67%) or living with a partner (30%) and had completed secondary school (91%) (Table 2). Only 40% of women were Australian born, however, 70% indicated English was their first language. Seventy percent of women were having their first baby.

**Table 2:** **Background characteristics of participants**

**Characteristic**	**n**	***%***
	**(n =108)**	
Age (years), mean (sd)	31.1	*4.7*
First baby (n = 107)	75	*70.1*
Marital status:		
Married	72	*66.7*
Living with partner	32	*29.6*
Have a partner but do not live together	3	*2.8*
Single	1	*0.9*
Highest education completed		
Completed degree or higher	57	*52.8*
Completed secondary school to year 12	42	*38.9*
Did not complete year 12	9	*8.3*
Country of birth (n = 103)		
Australia	43	*41.7*
India	10	*9.7*
New Zealand	5	*4.9*
Other* (includes 30 countries, each of which represent <5% of participants)	45	*43.7*
Length of time in Australia: (n = 56)		
Less than 5 years	37	*66.1*
5-10 years	3	*5.4*
More than 10 years	16	*28.6*
English as a first language	76	*70.4*
Smoked prior to pregnancy	20	*18.5*
Income (pretax household income per week, $AUD)		
<$650	12	*11.1*
$650-$999	18	*16.7*
$1000-$1399	23	*21.3*
$1400-$1999	26	*24.1*
>$2000	25	*23.2*
Did not answer	4	*3.7*

Women in this program were low risk at the time of recruitment, with no known medical problems that would prohibit early discharge, and 76% (81/107) did not have any complications recorded during pregnancy (Table 3). Of the 26 women with recorded pregnancy complications, most related to tests regarding fetal wellbeing. Births occurred between September 2007 and May 2008. The mean gestation at birth was 40 weeks (sd 2 weeks, range 26–42 weeks). Three quarters of the women (79/107) had a vaginal birth. Thirty-four percent (36/107) had labour and birth complications documented in their medical record and 17% (18/107) had postnatal complications documented.

**Table 3:** **Maternal and infant clinical outcomes**

**Outcome**	**n**	***%***
	**(n =107)**	
Pregnancy complications (e.g. impaired fetal wellbeing)	26	*24.3*
Type of birth		
Unassisted vaginal	61	*57.0*
Vacuum/forceps	18	*16.8*
Planned caesarean	7	*6.5*
Unplanned caesarean	21	*19.6*
Labour and birth complications*	36	*33.6*
Postpartum complications**	18	*16.8*
Birthweight (g) (mean, sd)	3454	*(544)*
Liveborn	107	*100.0*
Admission to special care or neonatal intensive care	14	*13.1*
Apgar score at 5 minutes (median, range)	9	*(6–10)*
Any breast milk feeding in hospital	105	*98.1*

*Postpartum haemorrhage (PPH) n = 26; precipitate labour n = 3; severe perineal trauma/hematoma n = 4, shoulder dystocia n = 2. **PPH n = 6; hypogalactica n = 4; endometritis n = 4; urinary tract infection n = 2.

All babies were liveborn and there were no neonatal deaths. The median five minute Apgar score was nine (range six to 10), and 15% of all babies (15/107) required some form of resuscitation (Table 2). The mean birthweight was 3454 g (sd 544, range 805-4630 g). Ten percent of babies (11/107) were admitted to the special care nursery (n = 8) or neonatal intensive care unit (n = 3) during their initial hospital stay. Nearly all babies (98%; 105/107) received at least some breast milk either directly from the breast or as expressed breast milk, although less than half (43%) were discharged feeding only from the breast.

### Individual plans for postnatal length of stay (as decided at 36 weeks)

Of the 108 participants, 103 women (95%) completed a plan of postnatal care at 36 weeks gestation. Of the five women who did not complete the plan; two contacted the recruitment midwife prior to their appointment indicating they preferred standard care, one did not have Medicare cover^a^ and opted to decline all home visits, one relocated out of the hospital domiciliary visiting area, and another woman had a preterm birth at 26 weeks, soon after recruitment. One woman gave birth (pre-term) before the eight week questionnaire was finalised. The remaining 107 women were sent questionnaires. Seventy-three of the 107 questionnaires were returned, a response fraction of 68%.

Of the women who completed the plan regarding their postnatal stay following a possible vaginal birth, 17% planned to leave by 12 hours postpartum, and 36% planned to stay 24 to 48 hours. Less than one in four women (23%, 24/103) chose to designate their length of stay in the case of a planned or unplanned caesarean birth. Of these 24 women, the majority preferred to stay three (38%) or four nights (38%). (NB: standard care in the public system at the time was 2.3 days following a vaginal birth and 3.9 days after a caesarean section [25] (Table 4).

**Table 4:** **Plans for preferred length of postnatal stay made at 36 weeks gestation**

	**n**	***%***
**Vaginal birth (n = 103)**		
Leave the same day of the birth (6–12 hours)	18	*17*
One night stay (12–24 hours)	46	*45*
Two nights stay (24–48 hours)	37	*36*
Option was not applicable (having a planned caesarean birth)	2	*2*
**Caesarean birth (n = 24)**		
One night stay (up to 32 hours)	1	*4*
Two nights stay ( up to 48 hours)	5	*21*
Three nights stay (48–72 hours)	9	*38*
Standard care (>72 hours)	9	*38*

In the questionnaire sent eight weeks after the birth women were asked if they considered that the concept of individualised postnatal care was introduced at an appropriate time during pregnancy, and 90% (60/67) of respondents agreed:

*I was starting to wonder about what would happen after the birth and the care I would receive. It was a great time to find out the options (ID1071, primiparous, 22 years, normal birth).*

*I hadn’t thought much about my hospital stay at this stage but discussing the program allowed me ample time to make a decision (ID1032, primiparous, 28 years, normal birth).*

The majority (76%; 54/71) of survey respondents indicated they felt ‘actively involved in planning for the postnatal period during pregnancy’.

### Length of postnatal stay

The median length of stay after birth for women in the pilot was 2.8 days (range 4 hours to 9.3 days) (mean 2.6 days, sd 1.5). Thirty-one percent (23/75) of the women having their first baby and 50% (16/32) of those having a subsequent baby stayed less than 48 hours (Table 5). Considering vaginal births only, 48% (38/79) were discharged less than 48 hours after the birth. Thirty-six percent of all women in the program were discharged less than 48 hours after the birth. This was twice the proportion of women being discharged prior to 48 hours in comparison to the general hospital population (18% during March and April 2008), although women in this study were at low risk of complications, compared to the mixed-risk general hospital population.

**Table 5:** **Postnatal length of stay by parity and method of birth**

	**≤ 12 hours**		**12-24 hours**		**24-48 hours**		**48-72 hours**		**>72 hours**		**>96 hours**	
	**n**	***%***	**n**	***%***	**n**	***%***	**n**	***%***	**n**	***%***	**n**	***%***
First baby (n = 75)	3	*4.0*	4	*5.3*	16	*21.3*	16	*21.3*	22	*29.3*	14	*18.7*
Subsequent baby (n = 32)	2	*6.3*	8	*25.0*	6	*18.8*	8	*25.0*	5	*15.6*	9	*9.4*
Vaginal birth (n = 79)	5	*6.3*	12	*15.2*	21	*26.6*	22	*27.9*	12	*15.1*	7	*8.9*
Caesarean birth (n = 28)	0	*-*	0	*-*	1	*3.6*	2	*7.1*	15	*53.6*	10	*35.7*
All births n = 107	5	*4.7*	12	*11.1*	22	*20.6*	24	*22.4*	27	*25.3*	17	*15.9*

### Planned versus actual length of hospital stay

Although the antenatal plan included a section where women could alter their plan after the birth, most women (79/103) left this section blank. Medical record data were used to determine length of stay, and women’s surveys used to ascertain if and why antenatal plans changed. Of those women who had a vaginal birth, 96% (76/79) had made an antenatal plan regarding their preferred length of stay after a vaginal birth. Of these, 62% (47/76) stayed longer than they planned, 11% (8/76) stayed a shorter time than they had planned, and 28% (21/76) stayed as planned. Of the women who had planned to stay 12 hours or less postpartum, 29% (5/17) achieved this, and 35% (12/34) of those planning to go home between 12 and 24 hours did so. Ten of the women who made a plan regarding their preferred length of stay after a caesarean birth actually had a caesarean birth. Of these women, three stayed longer than planned, five stayed for their planned length and two stayed for less time than planned.

### Factors to explain postnatal length of stay that was longer or shorter than planned

Of the women who stayed longer than planned following vaginal birth (n = 47), most extended stays appeared to be related to clinical complications. Postpartum haemorrhage (PPH) was the most common factor explaining the longer length of stay (23%; 11/47), with other possible factors including the woman being Group B Streptococcus positive (n = 10), having a third degree tear (n = 4), hematoma (n = 2), pre-eclampsia (n = 2), or incomplete placenta (n = 2); or infant admitted to the special care nursery (n = 2). However, from the medical records the exact reason(s) for extended length of stay was unclear and was not explicitly documented. The three women who stayed longer than planned after a caesarean birth also had at least one clinical complication that could explain the longer stay.

Although complications were often associated with stays longer-than-planned, this was not always the case. Nineteen percent (7/36) of the women with complications of labour and/or birth had a length of stay less than 48 hours. Two women with a precipitate labour stayed between 12 and 24 hours while four women who had a PPH and one woman who had mild shoulder dystocia stayed between 24 and 48 hours.

When reflecting on their length of stay in the postpartum survey, 61% (39/64) of women indicated that they modified their plans after the birth of the baby, with 90% (35/39) staying longer than planned and 10% (4/39) staying less time. Therefore many women did not achieve the length of stay they had planned during pregnancy (e.g. only 28% of those having a vaginal birth), and two thirds said that their plans changed after the birth of the baby. This needs to be taken into consideration in terms of whether women were happy with their length of stay, and whether they felt they had some control over what length of time they stayed in hospital.

### Home visits

Overall, of those women who had a vaginal birth and who had made an antenatal plan regarding their preferred length of stay and home visits after a vaginal birth, 62% had the appropriate number of home visits as per their plan (Table 6). Regardless of whether the length of hospital stay was as planned, only one woman had more visits than planned, and 28% received less home visits than they should have according to their antenatal plan. For women who had a caesarean (who had made an antenatal plan for this outcome), 7/10 received the planned number of visits at home.

**Table 6:** **Number of home visits compared to planned length of stay for vaginal births**

**Length of stay**	**Home visits as planned**		**More home visits**		**Fewer home visits**		**Unknown number of visits**	
	**n**	***%***	**n**	***%***	**n**	***%***	**n**	***%***
As planned (n = 21)	9	*43*	0	*0*	12	*57*	0	*0*
Longer than planned (n = 47)	36	*77*	1	*2*	3	*6*	7	*15*
Shorter than planned (n = 8)	2	*25*	0	*0*	6	*75*	0	*0*
**Total (all women who had a vaginal birth and had plan (n = 76))**	**47**	***62***	**1**	***1***	**21**	***28***	**7**	***9***

Only 37% (27/73) of respondents reported they were given an option for the *number* of postnatal home visits prior to being discharged home; however of these, 70% (19/27) reported receiving the number they requested. Similarly, only 41% of women (30/73) reported they were given an option for the *timing* of home visits. Notwithstanding this, women generally felt they received the ‘right number’ of home visits (53/72; 74%).

Forty-one women made comments in relation to the number or timing of visits, of whom 22% (n = 9) felt they had enough visits. Eight women indicated they would have preferred pre-arranged visit times.

*It would have been more helpful if there was an estimate of time, rather than having to wait all* day (*ID 1093, multiparous, 34 years, normal birth*).

[It] w*ould be better to be informed of a time frame of visits, e.g. 9–12, to be able to plan when to sleep (ID 1050, primiparous, 30 years, normal birth).*

The same number (n = 8) indicated they would have liked more visits:

*[The] midwives ‘discharged’ me early as I was healthy and baby fine, but I would have liked one more visit just for reassurance* (1051, *primiparous, 28 years, normal birth*).

*I would have liked two visits once at home as I felt pretty unsure about what I was doing* (1082, *primiparous, 31 years, normal birth*).

*I would like another visit about a week later* (1061, *primiparous, 30 years, normal birth*).

Two women reported receiving less visits than agreed; one received a phone call instead of the last visit and the other indicated that the hospital did not provide the visits due to demand:

*Two visits didn’t occur because I was told ‘we already have six visits booked for the next two days* (1076, *multiparous*, 36 years, *normal birth*).

Only one woman reported she had too many visits:

*I think midwives visiting every day is a bit much and unnecessary when it’s not the first child* (1094, *multiparous*, 28 years, *normal birth*).

### Women’s views of the program

Overall, women were supportive of the PinC program, with 88% (60/68) stating they would opt for the program in a future pregnancy and 87% (60/69) said they would recommend the program to family and friends.

#### Positives

One third of participants commented on the benefits of the program, including responses about being at home with their family, increased confidence with handling the baby at home and being able to get more rest.

*I have the ability to breastfeed now – and support of my partner at home during nights is fantastic. I’d rather be at home with my family (ID 1105, primiparous, 35 years, forceps birth).*

*I would be able to come home to my own kind of dish [food]. A more familiar environment with less visitors in the room. And I would be able to use my own bathroom and toilet (ID 1041, multiparous, 35 years, normal birth*).

Ten women liked the increased control and flexibility the program allowed, and these comments are typical.

*We found the flexibility really worked for us. Knowing that we could stay or go home when ready, with support, allowed us to do what felt right at the time with confidence. The visits at home were invaluable (ID 1009, primiparous, 30 years, normal birth).*

*It was fantastic for me. It allowed me the ability to try and use my skills before being told/shown by someone else. Overall a very positive experience that has increased my confidence. The people involved in the program made it wonderful (ID 1083, primiparous, 29 years, normal birth*).

*It made me feel as though I had some control over my care (ID 1051, primiparous, 29 years, normal birth).*

One said:

*It empowers people to make their own decisions and not rely on the system as gospel (ID 1037, multiparous, 32 years, normal birth).*

A number of the positive comments related to the domiciliary visits and the benefits of these.

*We … enjoyed having the midwife ourselves, one-on-one for the extended period of time. We thought that really worked well (ID 1009, primiparous, 30 years, normal birth).*

*I don’t think you will receive more help in the hospital. PINC = one on one support in your own environment (ID 1104, primiparous, 30 years, normal birth).*

A small number of participants (n = 7) would have preferred to spend less time in hospital.

#### Negatives

Five women’s comments indicated a lack of staff awareness regarding the program.

*[I] didn’t really get much info after the initial sign up. No one came to see me from the PinC program – still don’t really know what it did in my particular case (ID 1039, primiparous, 36 years, normal birth).*

*After the birth, the Pinc program was not discussed with me at all - perhaps I should have been reminded that leaving early was an option and that I would still have phone support and home visits (ID 1061, primiparous, 30 years, normal birth).*

Some women (n = 14) considered the PinC program to be suitable only for women who already had children, or who were well supported or who had had vaginal births.

*I suppose it’s not for everyone…I [wouldn’t] recommend it [to anyone] unless they are confident about the birth, probably for 2*^*nd*^*baby (ID 1013, multiparous, 29 years, planned caesarean birth).*

Only one woman wrote that she enjoyed the hospital stay and would not recommend the program saying,

*I found the staff at the hospital knew more about ways to help me and advice to give me when I needed it. I felt relaxed and healed better in the hospital. Having someone do the cleaning, preparing meals etc. gave me more time to rest and focus on the baby. I got all the help without feeling invaded in my own home (ID 1012, multiparous, 29 years, normal birth).*

One woman felt unable to make decisions on length of stay she might prefer given it was her first baby.

*As it was my first baby, I didn’t know what to expect about my hospital stay so I found it difficult to guess how long I would want to stay, and what I would need help with after I went home (ID 1061, primiparous, 30 years, normal birth).*

### Improving the PinC program

Thirty-nine women suggested improvements that could be made to the program. Of these, 17 suggested a need for improved communication between multidisciplinary providers as well as between staff and women, and eight made comments relating to improving integration of the program into practice, with more educational support, information, referrals or phone support.

*I found many midwives, doctors were unsure of the program while in hospital. I was ready to leave way before I was able to because I was chasing the midwife who did not know the process to follow. At most lead up appointments I had to tell them I was part of the program, they didn’t seem to know (ID 1093, multiparous, 34 years, normal birth).*

## Discussion

Although it could be argued that all care should be individualised, the reality in large tertiary settings is that a set number of days or hours is broadly applied to the postpartum hospital stay, and there is little room for deviation except if clinical requirements mean a woman needs to stay in hospital longer. Similarly, while continuity of care models enhance individualised care, the vast majority of women do not have access to continuity of midwifery care at the current time, thus it is important to explore other ways of providing individualised postnatal care. The PinC program intervention was antenatal preparation for individualised postnatal care with a shorter length of hospital stay traded for increased domiciliary visits. We examined the feasibility and acceptability of this for women participating in the pilot program and whether such an intervention would be amenable to testing in a randomised controlled trial.

Nearly 40% of the women approached agreed to participate in the pilot. While reasons for non-participation were not specifically sought, some women said they were already planning to have early discharge but did not want to participate in research, and others (e.g. multiparous women desiring early discharge) said they did not need any extra home visits and did not see any benefit in participating. This may account for the relatively low proportion of multiparous women in the pilot, and it is possible that more than 39% would be agreeable to the idea of a shorter hospital stay with supported early discharge if it was not part of a research project. Many women seemed unaware that early discharge was an option in general.

During the discussion of the individualised PinC plan late in pregnancy, two-thirds of the women indicated they would like a stay of less than 24 hours after a vaginal birth. The discussion regarding the individual postnatal plans took place with a member of the research team. However, it is unknown if the women were also having discussions with midwives and doctors about postnatal discharge. These results show that women are willing to discuss plans for the postnatal period during pregnancy, and to consider planning for supportive early discharge.

Individualised postnatal plans were printed on bright pink paper and placed in the front of the medical record in an effort to alert staff. In many cases it seemed that staff were unfamiliar with the PinC program despite several education sessions and the involvement of the management team and key clinicians on the project steering committee. The section on the plans that was supposed to be completed in the postnatal period confirming the plans or modifying them was left blank in over 75% of cases, suggesting that plans were not referred to after the birth. This is supported by women’s comments that staff appeared unaware of the plans. This level of missing data means we have no prospective data as to whether and how women changed their plans after birth; rather we have had to rely on their comments in the postal surveys. For the most part it is unknown whether changes were initiated by the women or by staff. Comments from the surveys indicated a high level of changes to plans in the early postnatal period, and a lack of staff knowledge about the program. It is likely that there were a combination of factors at play – a possible lack of staff engagement and awareness of the PinC program and the impact of complications of labour, birth and/or the postnatal period (which were experienced by a large proportion of the women).

A relatively high proportion of women (28%) received fewer home visits than planned. Of the 29 women (who had a vaginal birth) who had planned for a short hospital stay after a vaginal birth, and who stayed the same or less time than they planned, only 11 received the correct number of visits as per their postnatal plan (for example, a woman discharged home after one night should have received four visits as per the plan). The remaining 18 women all received fewer visits than the number indicated on the plan. There may have been issues around lack of staffing, limiting the ability to provide flexibility; a lack of knowledge of the program; or perhaps a philosophical view that women did not need so many postnatal home visits.

This apparent lack of staff engagement may have been largely due to a low awareness of the program. The number of women in the pilot represented a very small proportion (approximately 2%) of overall births in the hospital. Although multiple education sessions were conducted, the staff attending these may not have provided care for women in the program. Brief information about the PinC program and a contact phone number for the project coordinator was located on a cover sheet for the postnatal plan, however, only three staff contacted the coordinator for more information or inquiries about the plan. Therefore it may have been the case that unless women identified themselves as part of the PinC program, staff were unaware. It is possible that if such an intervention was introduced on a larger scale there might be more engagement across the staff, with greater awareness of the program and increased adherence in terms of length of stay and number of postnatal home visits. Other explanations for the low proportion of women who achieved their plans could be that the staff did not agree with the PinC program, i.e. there was some cultural resistance to the change; that there were inadequate resources available to enact the plans (e.g. midwives, cars); or that women chose to accept the care as it was provided, rather than asking to have care as per their plans, or chose not to adhere to their plans. It may also be that for some women, especially first-time mothers, planning for the postnatal period during pregnancy might be quite challenging, given they are planning for something about which they know very little.

Despite the numerous studies that have found that women are less than satisfied with postnatal care and that care should be more individualised and flexible [7,11,26] very few studies have implemented and evaluated new approaches to care. A ‘before and after’ study conducted in Sydney, Australia, explored the impact of a multifaceted intervention which aimed to improve the content and quality of postnatal care. Strategies included ‘one-to-one’ uninterrupted time between women and midwives, introduction of flexible breakfast arrangements, longer rest periods with minimal disturbance of women and where possible, provision of continuity of carer [26]. Although there were some positive changes (e.g. in strategies to improve rest for women), there were no significant differences in perceived quality of care, breastfeeding outcomes and maternal self-efficacy. The key strategy of ‘one-to-one’ time was not consistently implemented and like this study, the authors concluded that there is the potential for individualised care to impact on outcomes, but established organisational systems and priorities are difficult to change. A statewide review of hospital postnatal care conducted in Victoria, Australia also found that organisational structures such as standard postnatal documentation and fixed length of stay, may inhibit rather than support individualised care for women after childbirth [13].

This project was undertaken during a time of major change. Relocation of the hospital to a new site took place a month after the last birth in the program, and a restructuring of maternity services was undertaken and a new primary midwifery model introduced around the same time. The focus for staff over this period was on preparation for these changes, which may have resulted in the PinC program receiving less attention than it may have at another time of implementation. As was occurring across Australia, the increased number of births had a direct impact on bed availability and stretched resources. The PinC program took place as the ‘baby boom’ gathered momentum. Despite this, it may be that cultural factors were at play, such as midwives thinking a longer hospital stay is better for women. Although the women themselves seemed very open to the concept of the program, changing staff attitudes and hospital processes may be quite challenging. Involving midwives in the development of new interventions at the outset is an important consideration when midwives are the group required to ensure an intervention is implemented [27]. Understanding the PinC package from the midwives’ point of view is therefore critical, and the data from the focus groups and key stakeholder interviews (which will be reported elsewhere) will provide further insight into these issues.

## Conclusion

This feasibility study found that just over one third of low risk women were willing to participate in a study exploring individualised, planned early discharge followed by increased home-based postnatal care. Women were very positive about individualised postnatal care planning that commenced during pregnancy, however the proportion of women achieving their plans was much less than we had expected within a low risk population. The rate of obstetric intervention and postpartum complications was relatively high and staff perceptions of early discharge or lack of knowledge about the program may have contributed to the majority of women staying longer than planned.

Given these findings it is difficult to recommend that this particular approach would be achievable within the context of a randomised controlled trial, however similar approaches aiming to increase the individualised approach to postnatal care could be considered. Interventions based even more firmly within the current structures and with more potential buy-in from the clinical midwives providing home-based care warrant further investigation.

## Endnotes

^a^Medicare is Australia’s publicly funded universal health care system, operated by the government authority Medicare Australia. While Australia has reciprocal arrangements with some countries, citizens of most countries are not eligible for Medicare. Almost the entire population is covered by Medicare, with exceptions related to non-permanent residency status [28] Medicare Services [http://www.humanservices.gov.au/customer/subjects/medicare-services].
